# 2047 Mental illness public stigma, culture, and acculturation among Vietnamese Americans

**DOI:** 10.1017/cts.2018.93

**Published:** 2018-11-21

**Authors:** Mai Do, Jennifer McCleary, Diem Nguyen, Keith Winfrey

**Affiliations:** 1 Tulane University School of Public Health and Tropical Medicine; 2 LA CaTS, Tulane University School of Medicine

## Abstract

OBJECTIVES/SPECIFIC AIMS: Stigma has been recognized as a major impediment to accessing mental health care among Vietnamese and Asian Americans (Leong and Lau, 2001; Sadavoy *et al*., 2004; Wynaden *et al*., 2005; Fong and Tsuang, 2007). The underutilization of mental health care, and disparities in both access and outcomes have been attributed to a large extent to stigma and cultural characteristics of this population (Wynaden *et al*., 2005; Jang *et al*., 2009; Leung *et al*., 2010; Spencer *et al*., 2010; Jimenez *et al*., 2013; Augsberger *et al*., 2015). People with neurotic or behavioral disorders may be considered “bad” as many Vietnamese people believe it is a consequence of one’s improper behavior in a previous life, for which the person is now being punished (Nguyen, 2003). Mental disorders can also been seen as a sign of weakness, which contributes to ambivalence and avoidance of help-seeking (Fong and Tsuang, 2007). Equally important is the need to protect family reputation; having emotional problems often implies that the person has “bad blood” or is being punished for the sins of his/her ancestors (Herrick and Brown, 1998; Leong and Lau, 2001), which disgraces the entire family (Wynaden *et al*., 2005). In these cases, public stigma (as opposed to internal stigma) is the primary reason for delays in seeking help (Leong and Lau, 2001). Other research has also highlighted the influences of culture on how a disorder may be labeled in different settings, although the presentation of symptoms might be identical (see Angel and Thoits, 1987). In Vietnamese culture, mental disorders are often labeled điên (literally translated as “madness”). A điên person and his or her family are often severely disgraced; consequently the individuals and their family become reluctant to disclose and seek help for mental health problems for fear of rejection (Sadavoy *et al*., 2004). Despite the critical role of stigma in accessing mental health care, there has been little work in trying to understand how stigmatizing attitudes towards mental illness among Vietnamese Americans manifest themselves and the influences of acculturation on these attitudes. Some previous work indicated a significant level of mental illness stigma among Vietnamese Americans, and experiences of living in the United States might interact with the way stigma manifests among this population (Do *et al*., 2014). Stigma is a complex construct that warrants a deeper and more nuanced understanding (Castro *et al*., 2005). Much of the development of stigma-related concepts was based on the classic work by Goffman (1963); he defined stigma as a process by which an individual internalizes stigmatizing characteristics and develops fears and anxiety about being treated differently from others. Public stigma (defined by Corrigan, 2004) includes the general public’s negative beliefs about specific groups, in this case individuals and families with mental illness concerns, that contribute to discrimination. Public stigma toward mental illness acts not only as a major barrier to care, but can also exacerbate anxiety, depression, and adherence to treatment (Link *et al*., 1999; Sirey *et al*., 2001; Britt *et al*., 2008; Keyes *et al*., 2010). Link and Phelan (2001) conceptualized public stigma through four major components. The first component, labeling, occurs when people distinguish and label human differences that are socially relevant, for example, skin color. In the second component, stereotyping, cultural beliefs link the labeled persons to undesirable characteristics either in the mind or the body of such persons, for example people who are mentally ill are violent. The third component is separating “us” (the normal people) from “them” (the mentally ill) by the public. Finally, labeled persons experience status loss and discrimination, where they are devalued, rejected and excluded. Link and Phelan (2001) emphasized that stigmatization also depends on access to social, economic, and political power that allows these components to unfold. This study aims to answer the following research questions: (1) how does public stigma related to mental illness manifest among Vietnamese Americans? and (2) in what ways does acculturation influence stigma among this population? We investigate how the 4 components of stigma according to Link and Phelan (2001) operationalized and how they depend on the level of acculturation to the host society. Vietnamese Americans is the key ethnic minority group for this study for several reasons. Vietnamese immigration, which did not start in large numbers until the 1970s, has features that allow for a natural laboratory for comparisons of degree of acculturation. Previous research has shown significant intergenerational differences in the level of acculturation and mental health outcomes (e.g., Shapiro *et al*., 1999; Chung *et al*., 2000; Ying and Han, 2007). In this study, we used age group as a proxy indicator of acculturation, assuming that those who were born and raised in the United States (the 18–35 year olds) would be more Americanized than those who were born in Vietnam but spent a significant part of their younger years in the United States (the 36–55 year olds), and those who were born and grew up in Vietnam (the 56–75 year olds) would be most traditional Vietnamese. The language used in focus group discussions (FGDs) reflected some of the acculturation, where all FGDs with the youngest groups were done in English, and all FGDs with the oldest groups were done in Vietnamese. METHODS/STUDY POPULATION: Data were collected through a set of FGDs and key informant interviews (KIIs) with experts to explore the conceptualization and manifestation of mental illness public stigma among Vietnamese Americans in New Orleans. Six FGDs with a total of 51 participants were conducted. Participants were Vietnamese American men and women ages 18–75. Stratification was used to ensure representation in the following age/immigration pattern categories: (1) individuals age 56–75 who were born and grew up in Vietnam and immigrated to the United States after age 35; (2) individuals age 36–55 who were born in Vietnam but spent a significant part of their youth in the United States; and (3) individuals age 18–35 who were born and grew up in the United States. These groups likely represent different levels of acculturation, assuming that people who migrate at a younger age are more likely to assimilate to the host society than those who do at a later age. Separate FGDs were conducted with men and women. Eleven KIIS were conducted with 6 service providers and 5 community and religious leaders. In this analysis, we focused on mental illness public stigma from the FGD participants’ perspectives. FGDs were conducted in either English or Vietnamese, whichever participants felt more comfortable with, using semistructured interview guides. All interviews were audio recorded, transcribed and translated into English if conducted in Vietnamese. Data coding and analysis was done using NVivo version 11 (QSR International, 2015). The analysis process utilized a Consensual Qualitative Research (CQR) approach, a validated and well-established approach to collecting and analyzing qualitative data. CQR involves gathering textual data through semistructured interviews or focus groups, utilizing a data analysis process that fosters multiple perspectives, a consensus process to arrive at judgments about the meaning of data, an auditor to check the work of the research team, and the development of domains, core-ideas, and cross-analysis (Hill *et al*., 2005). The study was reviewed and approved by Tulane University’s Internal Review Board. RESULTS/ANTICIPATED RESULTS: Components of public stigma related to mental illness. The 4 components of public stigma manifest to different extents within the Vietnamese Americans in New Orleans. Labeling was among the strongest stigma components, while the evidence of the other components was mixed. Across groups of participants, Vietnamese Americans agreed that it was a common belief that people with mental disorders were “crazy,” “acting crazy,” or “madness.” “Not normal,” “sad,” and “depressed” were among other words used to describe the mentally ill. However, there were clear differences between younger and older Vietnamese on how they viewed these conditions. The youngest groups of participants tended to recognize the “craziness” and “madness” as a health condition that one would need to seek help for, whereas the oldest groups often stated that these conditions were short term and likely caused by family or economic problems, such as a divorce, or a bankruptcy. The middle-aged groups were somewhere in between. The evidence supporting the second component, stereotyping, was not strong among Vietnamese Americans. Most FGD participants agreed that although those with mental disorders may act differently, they were not distinguishable. In a few extreme cases, mentally ill individuals were described as petty thefts or being violent towards their family members. Similarly to the lack of strong evidence of stereotyping, there was also no evidence of the public separating the mentally ill (“them”) from “us”. It was nearly uniformly reported that they felt sympathetic to those with mental disorders and their family, and that they all recognized that they needed help, although the type of help was perceived differently across groups. The older participants often saw that emotional and financial support was needed to help individuals and families to pass through a temporary phase, whereas younger participants often reported that professional help was necessary. The last component, status loss and discrimination, had mixed evidence. While nearly no participants reported any explicit discriminatory behaviors observed and practiced towards individuals with mental disorders and their families, words like “discrimination” and “stigma” were used in all FGDs to describe direct social consequences of having a mental disorder. Social exclusion was common. Our older participants said: “They see less of you, when they see a flaw in you they don’t talk to you or care about you. That’s one thing the Vietnamese people are bad at, spreading false rumors and discrimination” (Older women FGD). One’s loss of status seemed certain if their or their loved one’s mental health status was disclosed. Shame, embarrassment, and being “frowned upon” were direct consequences of one’s mental health status disclosure and subsequently gossiped about. Anyone with mental disorders was certain to experience this, and virtually everyone in the community would reportedly do this to such a family. “You get frowned upon. In the Vietnamese culture, that’s [a family identified as one with mental health problems] the big no-no right there. When everybody frowns upon your family and your family name, that’s when it becomes a problem” (Young men FGD). This is tied directly to what our participants described as Vietnamese culture, where pride and family reputation were such a high priority that those with mental disorders needed to go to a great extent to protect—“We all know what saving face means” as reported by our young participants. Even among young participants, despite their awareness of mental illness and the need for professional help, the desire to avoid embarrassment and save face was so strong that one would think twice about seeking help. “No, you just don’t want to get embarrassed. I don’t want to go to the damn doctor and be like ‘Oh yeah, my brother got an issue. You can help him?’ Why would I do that? That’s embarrassing to myself…” (Young men FGD). Our middle-aged participants also reported: “If I go to that clinic [mental health or counseling clinic], I am hoping and praying that I won’t bump into somebody that I know from the community” (Middle-aged women FGD). Vietnamese people were also described as being very competitive among themselves, which led to the fact that if a family was known for having any problem, gossips would start and spread quickly wherever they go, and pretty soon, the family would be looked down by the entire community. “I think for Vietnamese people, they don’t help those that are in need. They know of your situation and laugh about it, see less of you, and distant themselves from you” (Older women FGD). Culture and mental illness stigma, much of the described stigma and discrimination expressed, and consequently the reluctance to seek help, was attributed to the lack of awareness of mental health and of mental health disorders. Many study participants across groups also emphasized a belief that Vietnamese Americans were often known for their perseverance and resilience, overcoming wars and natural disasters on their own. Mental disorders were reportedly seen as conditions that individuals and families needed to overcome on their own, rather than asking for help from outsiders. This aspect of Vietnamese culture is intertwined with the need to protect one’s family’s reputation, being passed on from one generation to the next, reinforcing the beliefs that help for mental disorders should come from within oneself and one’s family only. Consequently persons with mental health problems would be “Keeping it to themselves. Holding it in and believing in the power of their friends” (Middle-aged FGD) instead of seeking help. Another dimension of culture that was apparent from FGDs (as well as KIIs) was the mistrust in Western medicine. Not understanding how counseling or medicines work made one worry about approaching service providers or staying in treatment. The habit of Vietnamese people to only go see a doctor if they are sick with physical symptoms was also a hindrance to acknowledging mental illness and seeking care for it. Challenges, including the lack of vocabulary to express mental illness and symptoms, in the Vietnamese language, exaggerated the problem, even among those who had some understanding of mental disorders. It was said in the young men FGD that: “when you classify depression as an illness, no one wants to be sick,… if you call it an illness, no one wants to have that sort of illness, and it’s not an illness that you can physically see…” (Young men FGD). Another young man summarized so well the influence of culture on mental illness stigma: “Us Southeast Asian, like, from my parents specifically has Vietnam War refugees. I think the reason why they don’t talk about it is because it’s a barrier that they have to overcome themselves, right? As refugees, as people who have been through the war… [omitted]They don’t want to believe that they need help, and so the trauma that they carry when they give birth to us is carried on us as well. But due to the language barrier and also the, like, they say with the whole health care, in Vietnam I know that they don’t really believe in Western and Eurocentric medicine. So, from their understanding of how, like from their experience with colonization or French people, and how medicine works, they don’t believe in it” (Young men FGD). One characteristic of the Vietnamese culture that was also often mentioned by our FGD participants (as well as KIIs) was the lack of sharing and openness between generations, even within a family. Grandparents, parents, and children do not usually share and discuss each other’s problems. Parents and grandparents do not talk about problems because they need to appear strong and good in front of their children; children do not talk about problems because they are supposed to do well in all aspects, particularly in school. The competitiveness of Vietnamese and high expectations of younger generations again come into play here and create a vicious cycle. Young people are expected to do well in school, which put pressure on them and may result in mental health problems, yet, they cannot talk about it with their parents because they are not supposed to feel bad about school, and sharing is not encouraged. The Asian model minority myth and the expectations of parents that their children would do well in school and become doctors and lawyers were cited by many as a cause of mental health problems among young people. “Our parents are refugees, they had nothing and our parents want us to achieve this American Dream…. [omitted] It set expectations and images for us…. It was expected for all the Asians to be in the top 10, and for, like a little quick minute I thought I wasn’t going to make it, I was crying” (Yong men FGD). As a result, the mental health problems get worse. “If you’re feeling bad about something, you don’t feel like you can talk about it with anyone else, especially your family, because it is not something that is encouraged to be talked about anyway, so if you are feeling poorly and you don’t feel like you could talk to anybody, I think that just perpetuates the bad feelings” (Middle-aged women FGD). Acculturation and mental illness stigma Acculturation, the degree of assimilation to the host society, has changed some of the understanding of mental illness and stigmatizing attitudes. Differences across generations expressed in different FGDs indicated differences in perceptions towards mental illness that could be attributed to acculturation. For example, the young generation understood that mental illness was a health problem that was prevalent but less recognized in the Vietnamese community, whereas a prominent theme among the older participants was that mental illness was a temporary condition due to psychological stress, that it was a condition that only Caucasians had. Some of the components of public stigma related to mental illness seemed to vary between generations, for example the youngest participants were less likely to put a label on a person with mental health problems, or to stereotype them, compared to the oldest and middle-aged participants. This was attributed to their education, exposure to the media and information, and to them “being more Americanized.” However, there was no evidence that acculturation played an important role in changing the other components of public stigma, including stereotyping, separating, and status loss and discrimination. For example, the need to protect the family reputation was so important that our young participants shared: “If you damage their image, they will disown you before you damage that image” (Young men FGD). Young people, more likely to recognize mental health problems, were also more likely to share within the family and to seek help, but no more likely than their older counterparts to share outside of the family—“maybe you would go to counseling or go to therapy, but you wouldn’t tell people you’re doing that” (Young women FGD). The youngest participants in our study were facing a dilemma, in which they recognized mental health problems and the need for care, yet were still reluctant to seek care or talk about it publicly because of fears of damaging the family reputation and not living up to the parents’ expectations. Many young participants reported that it actually made it very difficult for them to navigate mental health issues between the 2 cultures, despite the awareness of the resources available. “I think it actually makes it harder. Only because you know to your parents and the culture, and your own people, it’s taboo, and it’s something that you don’t talk about. Just knowing that you have the resources to go seek it… You want advice from your family also, but you can’t connect the appointment to your family because you’re afraid to express that to your parents, you know? So I think that plays a big part, and knowing that you are up and coming, but you don’t want to do something to disappoint your family because they are so traditional” (Young men FGD). Some participants felt more comfortable talking about mental health problems, like depression, if it was their friend who experienced it and confided in them, but they would not necessarily felt open if it was their problem. Subtle cultural differences like this are likely overlooked by Western service providers. One older participant summarized it well “They [the young generation] are more Americanized. They are more open to other things [but] I think that mental health is still a barrier.” DISCUSSION/SIGNIFICANCE OF IMPACT: This study investigated how different components of public stigma related to mental illness manifest among Vietnamese Americans, a major ethnic group in the United States, and how acculturation may influence such stigma. The findings highlighted important components of public stigma, including labeling and status loss, but did not provide strong evidence of the other components within our study population. Strong cultural beliefs underlined the understanding of mental health and mental illness in general, and how people viewed people with mental illness. Several findings have been highlighted in previous studies with Asian immigrants elsewhere; for example, a study from the perspectives of health care providers in Canada found that the unfamiliarity with Western biomedicine and spiritual beliefs and practices of immigrant women interacted with social stigma in preventing immigrants from accessing care (O’Mahony and Donnelly, 2007). Fancher *et al*. (2010) reported similar findings regarding stigma, traditional beliefs about medicine, and culture among Vietnamese Americans. Acculturation played a role in changing stigmatizing attitudes as evidenced in intergenerational differences. However, being more Americanized did not equate to being more open, having less stigmatizing attitudes, or being more willing to seek care for mental health issues. Consistent with previous studies (Pedersen and Paves, 2014), we still found some level of stigma among young people aged 18–35, although some components were lessened with an increased level of acculturation. There was also a conflict among the younger generation, in which the need for mental health care was recognized but accessing care was no easier for them than for their parent and grandparent generations. The study’s findings are useful to adapt existing instruments to measure stigma to this population. The findings also have important program implications. One, they can be directly translated into basic supports for local primary and behavioral health care providers. Two, they can also be used to guide and inform the development and evaluation of an intervention and an additional study to validate the findings in other immigrant ethnic groups in the United States. Finally, based on results of the study, we can develop a conceptual framework that describes pathways through which social, cultural, and ecological factors can influence stigma and the ways in which stigma acts as a barrier to accessing mental health care among Vietnamese Americans. The guiding framework then can be validated and applied in future programs aimed to improve mental health care utilization among ethnic minorities.

